# Surface Treatment of the Dental Implant with Hyaluronic Acid: An Overview of Recent Data

**DOI:** 10.3390/ijerph18094670

**Published:** 2021-04-27

**Authors:** Gabriele Cervino, Agron Meto, Luca Fiorillo, Alessandra Odorici, Aida Meto, Cesare D’Amico, Giacomo Oteri, Marco Cicciù

**Affiliations:** 1 Department of Biomedical and Dental Sciences, Morphological and Functional Images, University of Messina, 98100 Messina, Italy; gcervino@unime.it (G.C.); cdamico@unime.it (C.D.); goteri@unime.it (G.O.); mcicciu@unime.it (M.C.); 2 Department of Implantology, Faculty of Dentistry, University of Aldent, 1000 Tirana, Albania; agronmeto@yahoo.com; 3 Multidisciplinary Department of Medical-Surgical and Odontostomatological Specialties, University of Campania “Luigi Vanvitelli”, 80121 Naples, Italy; 4 Laboratory of Microbiology and Virology, School of Doctorate in Clinical and Experimental Medicine, University of Modena and Reggio Emilia, Via G. Campi 287, 41125 Modena, Italy; odorici.alessandra@gmail.com; 5 Department of Dental Therapy, Faculty of Dental Medicine, University of Medicine, 1005 Tirana, Albania; aidameto@yahoo.com

**Keywords:** dental implant, surface treatment, hyaluronic acid

## Abstract

Recently, interest has grown by focusing on the evaluation of a molecule already produced in the human body such as hyaluronic acid (HA), as an application to the surface of the titanium implant. Its osteo-conductive characteristics and positive interaction with the progenitor cells responsible for bone formation, consequently, make it responsible for secondary stability. The aim of this work was to analyze the various surface treatments in titanium implants, demonstrating that the topography and surface chemistry of biomaterials can correlate with the host response; also focusing on the addition of HA to the implant surface and assessing the biological implications during early stages of recovery. Used as a coating, HA acts on the migration, adhesion, proliferation and differentiation of cell precursors on titanium implants by improving the connection between implant and bone. Furthermore, the improvement of the bioactivity of the implant surfaces through HA could therefore facilitate the positioning of the dental prosthesis precisely in the early loading phase, thus satisfying the patients’ requests. It is important to note that all the findings should be supported by further experimental studies in animals as well as humans to evaluate and confirm the use of HA in any field of dentistry.

## 1. Introduction

Implantology is a branch of dentistry that has made countless advances over the past decades. The basic principles of integrated osteo-implantology have undergone significant changes that are modifying the therapeutic modalities faced by the profession [1,2]. Obtaining excellent results in oral rehabilitation, solving aesthetic, functional and psychological problems in both partial and total edentulism [3,4,5,6,7].

In various clinical situations, thanks to all the results obtained, it is possible to anticipate loading times, satisfying expectations and consequently increasing the patients’ confidence in these treatments. Implant-supported prostheses, actually, have significant advantages over adhesive or mucous ones, because the titanium implant solicits the bone stimulating the maintenance of its vertical and horizontal dimensions in a similar way to natural teeth [8,9].

Moreover, given the progress already achieved and the extensive possibilities for improvement, there are still many areas of research in the implantology field aimed at optimizing surgical and prosthetic procedures [10]. During the growth of clinical processes, technologies and their products, the ongoing interaction between implant research, prosthetics and the industrial field has led to the inclusion of increasingly versatile and valuable components to be utilized in oral rehabilitation [11]. Recently, the literature has focused on the dental implant surface, both in terms of the osseointegration rate and the amount of bone-implant bonding. Basically, there are numerous commercial titanium implants whose surfaces are subjected to various treatments, able to modify the surface features in favor of a greater osteoconductivity. From the smooth implant, used as the first prototype, it was moved to implants characterized by macro and micro topography by means of sandblasting procedures with an aluminum oxide jet or titanium microparticles; high pressure and high-temperature titanium plasma spray (TPS) procedures; high-temperature calcium and phosphate granule melting processes; combined sandblasting and etching processes with strong and weak acids (ALS) [12]; coating processes with calcium and sodium vitreous phosphosilicates (bioactive glass) [13].

Current efforts are focused on evaluating a material already produced within the human body such as hyaluronic acid as an implementation on the titanium implant surface. The rational use of this glycosaminoglycan, part of the extracellular matrix, arises from its characteristics of osteoconductivity and positive interaction with the progenitor cells responsible for bone formation and, consequently, responsible for secondary stability. Studies demonstrate that the topical application of a HA gel in the peri-implant pocket and around implants with peri-implantitis may reduce inflammation and crevicular fluid IL-1β levels [14,15,16,17,18,19,20].

The aim of this study was to review the current literature concerning different implant surface treatments and to investigate if (a) surface topography and chemistry can be related to host response; (b) the possible use of HA on implant surface; (c) to evaluate biological procedures in early stages of healing process.

## 2. Osseointegration

Osseointegration is defined as the direct, structural and functional connection between a vital bone and the implant surface without the interposition of soft tissue. Likewise, the characteristics needed to achieve a rigid osseointegration were determined by Brånemark, Albrektsson et al. in the early eighties, with studies setting out some rules to follow in relation to [21,22]:

- biocompatibility,
- form,
- implant surface,
- implant site,
- surgical technique,
- loading conditions.

However, it is not right to consider osseointegration as a prerogative of any accentuated implant device, considering that it does not have the same result; as in bone of low quantity and quality as reported by Albrektsson and Johansson [15]. Cook et al. [23] examined, in one of their works, the variants that oriented the bone apposition on the implant surfaces of all the considered parameters, where only the surface characteristics showed a dominant role in implant integration. In particular, a change in the surface characteristics of the implants can lead to better clinical outcomes, i.e., surface properties. Essentially, osseointegrated implants play a crucial role in the development dynamics of osseointegration, and for this reason, the study of implant surfaces is still current. Both the surface topography and the chemical composition are key factors. This was also confirmed by Kononen et al. [24] emphasizing that surface topography can directly influence cell structure, orientation, function and proliferation. While, according to Kasemo and Lausmaa et al. [25,26,27,28], the chemical composition influences the biocompatibility since the interaction with biological tissues is related to the properties of the outermost atomic layer of the implant surface. Importantly, both topographical and chemical approaches are required to fully understand the surface characteristics of a structure. Another determining factor is the level of surface contamination, where titanium, due to the oxide surface, is considered not-toxic and therefore highly biocompatible; however, the amount of the oxide layer surrounding it and its composition are mainly related to the manufacturing conditions. The implant success can be directly linked to the chemical elements present on the outside following a surface treatment [12]. Several in vitro studies have also demonstrated that sterilization protocols can influence cell behavior. Moreover, to better understand the influence of the implant surface on osteointegration, it is necessary to know the bone changes after implant insertion, the general properties of titanium and the characteristics of surface treatments.

## 3. Bone Modifications after Implant Placement/Insertion

The placement of an implant fixture inside the bone region must be considered damage of the organism’s integrity. Around the implant, there is always an empty micrometric space, in which complex biological phenomena are present, where from the beginning can be manifested an ischemia of the tissues with necrobiosis on the bone side [29]. The increased vascular permeability in the intervention area results in the transfer of undifferentiated mesenchymal cells to cover the cavity between the bone and implant surface (vascular cell migration and colonization). After the first four days, cell differentiation and organization of peri-implant tissue take place to allow the removal of cellular and bone debris from the necrosis removed by macrophages to begin subsequently the repair phase. The formation of the new bone follows all the phases that characterize the direct ossification, including here the osteoblasts arrival, deposition of the osteoid tissue, formation of immature bone with interlaced fibers. During the sixth week, the primitive bone is progressively reabsorbed and then replaced by mature lamellar bone; this process leads to the formation of bone around the inserted implant [30,31].

## 4. General Properties of Titanium

According to a widespread classification, implantable materials are divided into biotolerant, bioinert and bioactive. Biotolerant materials are characterized by a type of healing called “distant osteogenesis,” where the ions released by the material inserted into the host interfere with the cell metabolism and lead to the production of fibrous connective tissue. Then, bioinert materials do not release ions or harmful substances that can affect the cell metabolism and do not stimulate adverse tissue reactions, therefore a “contact osteogenesis” occurs without any interposition of connective tissue. Bioactive materials produce a favorable response, facilitating bone deposition thereby establishing chemical bonds with tissue components such as hydroxyapatite or stimulating cellular activity. While, due to their mechanical characteristics and the level of current technologies, they can only be used as coatings, because they are unable to support the forces discharged on a dental element [32]. Various materials have been tested in the construction of dental implants, some of which are no longer used; while implantable materials now considered biocompatible are commercially pure titanium (cp Ti), titanium alloys such as (Ti-6Al-4V), Al_2_O_3_-based ceramics, hydroxyapatite and zirconia. Titanium is a well-known metal in the dental field, where its physical, mechanical and biological characteristics, in fact, allow it to be universally used for the production of crowns, bridges, prostheses and implant systems. Moreover, it has a relative density of 4.5 g/cm, reaching melting at 1677 °C, boiling at 3277 °C and its thermal conductivity is leveled downward (22 Wm^−1^ K^−1^). Additionally, it has a strong mechanical resistance (breaking load of 730–950 N/cm^2^) and a consequent elongation of 12% breakage. The elastic modulus is low enough and very similar to that of bone (110.000 N/cm^2^) [33,34,35]. Typical characteristics of this metal are the high resistance of corrosion and the high degree of biocompatibility. While in nature, it appears essentially as dioxide (TiO_2_) in three polymorphic modifications such as rutile, anatase and brookite. The solid metal titanium is stable in air but with oxygen concentrations above 35%, and on a cold metal surface, obtained after the cutting test, it oxidizes rapidly. According to international standards of the American Society for Testing and Materials (ASTM) and the International Organization for Standardization (ISO), titanium is classified as follows in Table 1. Despite this classification, these alloys could present impurity (Table 2) [36].

The increase of titanium degree is directly proportional to the impure elements present (O_2_, Fe) that lead, on the one hand, to the improvement of the mechanical properties (such as modulus of elasticity and hardness), and on the other, to the reduction of the osseointegration capacity. The most widely used titanium in the medical field is grade II, which manages to best combine the appropriate physical properties with excellent corrosion resistance and biocompatibility characteristics. These qualities are mainly related to the formation of a rutile film (TiO_2_) and other oxides on its surface (TiO, Ti_2_O_3_, Ti_3_O_4_); wherein the film in question is homogeneous and inert, adherent and persistent and, if removed, is automatically reformed in a tiny fraction of a second.

In the implant-prosthetic field, it is also usual to use titanium of grade V. The latter is an alloy formed from a titanium base, with the presence of Al 6%, which helps to increase the hardness and decrease the specific weight, thus giving a greater modulus of elasticity; while the V 4% lowers the thermal conductivity (about 5% less) and improves resistance to fatigue and wear. Interestingly, the reduction reaction of tetrachloride with sodium and magnesium is used to produce titanium industrially. Once it reaches the spongy shape, it is melted in graphite containers, using induction furnaces and argon atmosphere, or voltaic arc in cooled copper tubes. During mechanical processing, this metal is constantly exposed to the atmosphere and other substances such as coolants and lubricants. Despite this, contact with air will cause a rapid generation of a titanium oxide layer of about 10 Ä, in less than a thousandth of a second, so within a minute, the thickness will increase to about 50–100 Ä. In fact, the sterilization process carried out by ultrasound or autoclave also plays a fundamental role in increasing the thickness of the oxide [37].

Among the oxides available on the surface, TiO_2_ is the most stable and present on the surface of titanium and its alloys and, consequently, this oxide negatively charges the implant, increasing the affinity for the different biomolecules. During the various phases of implant insertion, the oxide can be removed by the action of mechanical insertion; to reform itself instantly having an important role for osseointegration since it has the ability to prevent corrosion thanks to the high chemical stability and the diffusion of metal ions inside the tissues, giving it a high degree of biocompatibility [38].

## 5. Surface Treatments

The surface treatments of the implants are intended to generate a biologically active surface, which allows to improve the osseointegration between the implant and bone tissue. The presence of micro-treatments on the surface of the fixture allows an increase of the tensile strength and torsion of the implant [39,40].

Different authors have shown how macrophages, epithelial cells and osteoblasts have exhibited a high trophism against rough surfaces, i.e., rugophilia [41,42,43,44,45]. Several hypotheses have been put forward regarding different micro-treatment techniques of the external surface, with the aim of obtaining an ideal roughness that improves osseointegration. Sandblasting with alumina (Al_2_O_3_), TPS and hydroxyapatite are the most common treatments. Strong etching techniques have recently been proposed combined with traditional sandblasted (Sand-blasted Large-grid 21 Acid-etched: SLA). Among other methods, we have the coating with bioactive material such as biocompatible glass. The different types of implant surfaces can be classified into two main groups (smooth and rough treated surface dental implants) [37].

### 5.1. Smooth Implants

The “smooth” systems can be electropolished or machined (turned). While for electropolished installations, the raw surface undergoes an electrochemical treatment by immersion in an electrolytic solution in which the current passes and where the other electrode is predominantly platinum. In this way, it is possible to obtain roughness values of 10 nm. Therefore, for machined or turned systems, the surface undergoes a mechanical turning; microscopically it appears shiny and smooth, where the scanning microscope shows rotating streaks with minimal roughness. Several experimental and clinical studies have shown the predominance of rough-surface implants compared to smooth-surface ones in terms of the speed of the osseointegration process, the percentage of contact in the bone interface implantation and resistance to torsion tests. The evolution of the titanium surfaces used has allowed a partial halving of the healing time and prosthetic loading of the implants, passing from the initial six months to three-four weeks thanks to the considerable hydrophilicity and consequently to the greater ability to attract organic fluids such as blood. According to Johnson Davies’ work, has been indicated that in the case of rough implants, the platelet activation increases resulting in a greater presence of molecular mediators, increases the retention of fibrin on the surface of the implant thus determining a more stable network of fibrin already present in the early stages of the osseointegration mechanism and fundamental for the bone-implant connection. Thus, in the case of smooth implants, the number of platelets is lower, the fibrin is attacked less tenaciously and pericyte cells cannot reach the implant surface, giving rise to a distant osteogenesis and not to an osteogenesis by contact that occurs with rough surface implants [46].

### 5.2. Rough Surface

#### 5.2.1. Sandblasting

Sandblasting is a titanium modification technique that gives the same surface roughness, allowing an overall improvement of the implant biomechanical characteristics. This is carried out by special machines, those of sandblasting, which use a high-pressure jet containing certain substances such as aluminum oxide or titanium oxide. The jet, directed on a clean surface, erodes it slightly, creating grooves with a diameter of about 5–20 µm and thus making it rough; therefore, this treatment positively influences the primary stability as macrophages, epithelial cells, and osteoblasts show roughness for this layer. However, the modification of the surface should not interfere with the biocompatibility of the material. It has been shown that sandblasting with aluminum oxide particles with a diameter between 100 and 150 microns favors better osseointegration; the irregular surface increases the osteoconductivity of the metal, otherwise inert, favoring the adhesion and the activity of the osteoblasts. As a result, healing of the bone-implant interface is faster [47].

#### 5.2.2. Surfaces TPS

Another method of surface treatment involves coating with TPS or titanium powders, usually in the form of Ti-6Al-4V alloy. The plasma spraying process is carried out using a plasma burner with a voltage arc; the latter is produced between a copper anode and a cooled tungsten cathode. Across this system, titanium powder with a particle size between 50 and 100 µm, lies on the body of the fixture on which is placed. The coating obtained reaches a thickness of 0.5–2 mm and an average diameter roughness of around 200 µm. The treatment performed with TPS is therefore able to increase the available surface in close contact with the bone structure in a higher way superior to normal sandblasting, with a quality anchoring bone. The disadvantage of this technique is the poor contamination control, the possibility of a separation of particles and diffusion of metal ions from the implant surface [48].

#### 5.2.3. Surfaces Coated with Hydroxyapatite

Hydroxyapatite is a substance present in the composition of the mineralized structures of the body; in addition to being autologous, it can artificially reproduce with porous or dense consistency, granular powder and pre-formed blocks. Thus, it forms a chemical bond with the bone (titanium, in fact, exploits a mechanical bond, benefiting from superficial micro-actions) and does not cause local or systemic toxicity and phlegmosis [49,50,51].

The insufficient mechanical properties are therefore optimized thanks to its use as a coating of titanium surfaces. It is composed of calcium and phosphate granules joined in the laboratory through high-temperature processes, i.e., 125 °C. The reason why it is used is that the high concentration of phosphate and calcium favors the migration and maturation of the osteogenic cells that produce the matrix bone allowing a faster mineralization in contact with the surface. The processing technique is similar to that of obtaining TPS, with a decrease of the crystallinity of this material (ranged from 5% to 60–70%). Several studies have shown that the HA coating can lead to an improvement in clinical results; actually, there is a higher bone-screw contact, a greater amount of bone tissue and the absence of osteolysis areas among the threads of the screws covered with HA, compared to those without coating [51].

#### 5.2.4. Sandblasted and Etched Surfaces

**Figure 1:**
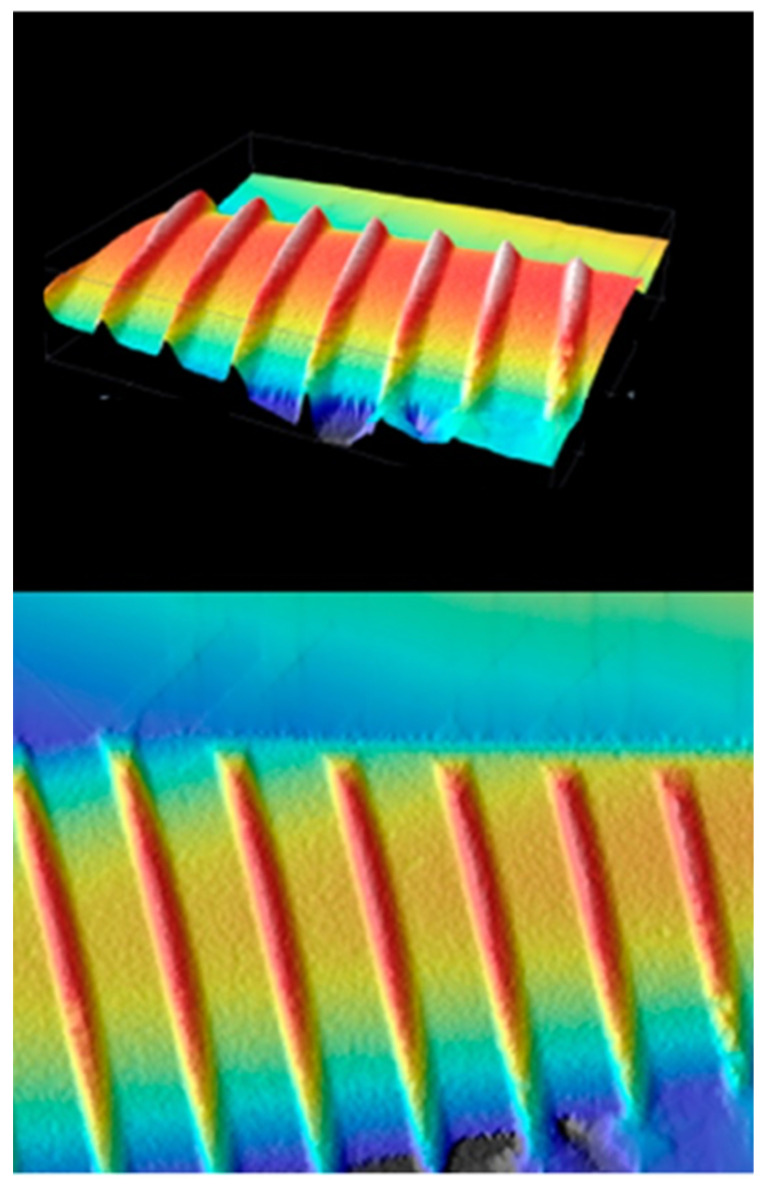
SLA surface confocal microscopy.

In 1990 was proposed a new surface-coat called SLA. The latter is a sandblasted surface with coarse-grained sand (250–500 nm in diameter), washed inside an ultrasonic tank with deionized water, subsequently dried, inserted then in a thermostat solution of sulphuric and hydrochloric acid, and finally rinsed and dried with hot air. This type of surface treatment determines a macro-rough layer related to the action of the sandblaster; simultaneously the action produced by the acid generates the creation of micro-alveoli (micro-roughness). This particular surface characterization assimilates the SLA implants to something other than a simple coating, which is the surface structure of TPS implants. Stain and McCollin confirm that insertion of TPS implants in the patients’ jaws, to obtain a stable osseointegration in bone class 1-2-3 according to Mish, takes three months, while in bone class 4 the times are lengthened from four to six months. Instead, implants with biomimetic characteristics are inserted, such as SLA implants, the time is significantly reduced to about six weeks in classes 1-2-3 and to twelve weeks for class 4, as a possibility to favor the proliferation and differentiation of cells with osteogenic potential in shorter times and with better performance (Figure 1) [12,52].

#### 5.2.5. Surfaces Coated with Bioactive Glass

Biologically active glasses belong to the family of glasses which, biocompatibility in vitro and in vivo, lack of inflammatory and toxic processes, in the presence of osteogenic precursors make it possible for osteocondunctive ability to promote a special biological bond in the contact areas of the glass within the bones. They are mainly composed of calcium phosphosilicates and sodium vitrosis; 45% of the mass consists of Si_2_O (vitrifier), 24.5% of N_2_O (flux), 24.4% of CaO (stabilizer) and the remaining percentage of P_2_O_2_ (binder) [53,54].

Bioactive glasses, unlike traditional glasses, are hydrolytically unstable; the biological activity is related to a phenomenon of surface hydrolytic degradation with the release of ions-sodium, calcium, silicon, phosphorus, potassium, phosphate, capable of creating a layer with high osteoconductive activity guaranteed by hydroxyapatite crystals that favors the migration and proliferation of osteoblasts. The result of such activity is the resorption of the glassy state and its replacement with newly formed bone tissue within a few months.

According to Anderson’s classification, inert glasses that lead to the formation of the fibrous tissue interface are included in class A. In classes B and C, glasses are very and fairly soluble; however, they are not able to form a stable bond with the bone tissue; while in Classes D and E, glass panes with controlled reactivity, capable to form a more or less consolidated bond with the bone tissue. In vitro and in vivo tests have shown positive characteristics of a coating obtained as a bioactive sprayed glass coating bioactive on titanium alloy (by means of a plasma-spray process); the result is the complete degradation of the glass layer and its replacement with the bone tissue in direct contact with the titanium implant.

#### 5.2.6. Trademark Surfaces: Tioblast, Osseotite, and TiUnite

The Tioblast technique uses an ablative and compacting method. The process aims to improve the characteristics of titanium through sandblasting produced with titanium dioxide granules under controlled conditions, producing a plastic deformation of the surface and generating holes of regular size and shape (1–5 microns).

This guarantees an interconnection force between bone and implant of three times greater than that obtainable with a standard implant, with an increase in surface area of about 15%.

The Osseotite surface is characterized by the subdivision in two portions, one smooth and one mordant/etching (Hcl/H_2_SO_4_). Studies show that acid etching produces a more uniform surface and therefore significantly greater bone-implant contact than machined surfaces.

The anodic oxidation, characteristic of Tiunite, consequently determines a gradual and controlled increase of the superficial layer of TiO_2_ and of the surface roughness in the apical direction. The surface includes a porous structure which, together with the increasing roughness, causes an increase in the surface. The results show significantly greater bone-to-implant contact than with turned or machined surfaces, greater torque removal and therefore greater stability.

### 5.3. New Surface Treatment That Does Not Alter Roughness and Favors Osseointegration: Hyaluronic Acid

Biochemical methods of implant surface modification endeavor to utilize the current knowledge of biology and biochemistry of cell function and differentiation.

Surface modification is performed to influence tissue responses; the purpose of tissue modification is to immobilize proteins, enzymes, or peptides on the surfaces of devices in order to induce specific tissue responses.

In 1904, Pfaundler hypothesized that calcium binding was an important step during bone calcification and that some unknown components were responsible. It was later discovered that gags play an important role and that HA improves the proliferation and growth of hydroxyapatite crystals. HA covalently bonded to the titanium implant surfaces significantly increases bone growth and results in greater maturity for the interfacial bone.

## 6. Studies on Surface Modifications of Dental Implants Using HA

The new strategy for improving the bone-implant interface is that of immobilization of matrix components on extracellular Ti, peptides or enzymes such as type I collagen to favor osseointegration by facilitating the adhesion of osteoblasts on implant surfaces.

The rational use of HA is linked to its composition, being one of the important glycosaminoglycans in the cellular matrix synthesized by fibroblasts, synovicytes and chondrorocytes; the same shows a significant reduction of inflammation during wound healing, favoring cell proliferation, re-epithelialization and scar reduction [55].

Type I collagen is the ideal candidate for tissue engineering grafts as it has a weighted blood compatibility and osteoblastic adhesion, decisive differentiation in bone mineralization, bone healing, enhances and secretion of extracellular matrix. This molecule, by increasing the wettability of the surface with which it is in contact, allows a better organization of the blood clot and the consequent cascade healing phenomena directly related to the presence of the same [55].

Recent scientific studies in different laboratories in New Zealand, examining rabbits as guinea pigs, have analyzed the effects of HA able to determine on the healing of different parts of the body, in particular the jaw, the tibia and the femoral knee; thus, highlighted positive feedback, although limited by the small number of samples taken, about the effectiveness of this type of implant surface modification [56,57,58,59].

### 6.1. Evaluation of TNF-α

The study of Hasan et al. [60] aimed to evaluate the effects of HA on the bone-implant interface by immunohistochemical estimation of tumor necrosis factor (TNF-α) in two groups of fifteen rabbits each. In both groups (experimental and control) sixty implants were placed in the tibias of the rabbits; where in the right tibia the uncoated implant was inserted, in the left the experimental one coated with 0.1 mL of HA gel). All rabbits, in groups of ten, were sacrificed one, two and four weeks after implant insertion surgery. Immunohistochemical tests were performed to evaluate the TNF-α expression on both groups at all healing intervals (Table 3) [60].

The highest mean value of positive TNF-α expression was found for osteoblasts and osteocytes at week four for the experimental group; indeed, osteoclasts in the second week with no significant differences between the experimental and control groups. TNF-α has an inhibitory effect during the various differentiation stages of osteoblasts and can act on the precursor of the same by increasing cellular differentiation of stem cells. The increased positive expression of TNF-α in the experimental group in the early stages of post-surgical healing indicates an acceleration of osseointegration for HA-coated implants [39].

### 6.2. Histomorphometric and Histochemical Analysis

The purpose of the studies on the HA effects in the dental implant integration was to histologically and histometrically evaluate the jaws of ten rabbits; in which two implants were inserted in front of the standard control plant and behind the implant integrated with hyaluronic acid gel. The results were discussed two months after surgery [39].

Three rabbits from the control group did not have osseointegration of the implant and were therefore excluded from the experiment; meanwhile, on the remaining rabbits, at the time of sacrifice, osseointegration was present in both groups but no signs of infection were assessed in both groups [56,57,58,59].

Histologically, the newly formed bone tissue was visible in both groups with a slight prevalence of osteoid tissue and new bone in the experimental group; but without significant differences as shown in Table 4.

Although there was no significant difference, the group treated with the experimental technique responded positively to the study issue.

### 6.3. Effects of Collagen on Healing and Bone Formation in a Rabbit Model as a Coat on Ti Implants

Further analyzes on this topic aimed to evaluate the bone-implant interface of thirty-six implants placed in the femoral knee joints of eighteen rabbits. In addition, these were performed using X-ray photoelectrons spectroscopy (XPS), atomic force microscopy (AFM), micro-TC and histologically fifteen, thirty and sixty days after surgery [39,61].

#### 6.3.1. Surface Analysis Using XPS

The comparison between the control group, sandblasted and etched implants, and experimental one with implants coated with type I collagen, showed that the implant surface in the first case is covered by an oxide layer of 4 mm thick (TiO_2_) 33% of pure titanium. The surface contamination due to the adsorption of hydrocarbons present in the atmosphere introduces presence of carbon on the surface; in the second case the titanium signal almost disappeared, the ratios O/Ti and C/Ti increased and the nitrogen rose up to 14%. The presence of this organic molecule confirmed that collagen was in the titanium surface [62].

#### 6.3.2. Surface Analysis Using AFM

The topography of both samples presented the typical micro-angularity due to acid etching. However, no evidence of organic layer was observed; although the vertical resolution was sub-nanometric and the microscopic analysis showed no collagen molecules on the implant’s surface. The roughness index Sdr demonstrated an increase in percentage of effective surface compared to the geometric one, which resulted from 88 to 7 for the control sample and from 85 to 3 for the experimental sample [39,63].

Therefore, there are no significant differences between these two groups. This result confirmed that the process did not involve the deposition of a thick, but nanometer-sized layer. This observation also suggested that the differences between the control and the experimental groups were not related to the surface modification at the topographical level, but rather at the chemical one.

#### 6.3.3. Micro-TC Evaluation

X-rays showed new bone in close contact with the implant surface in both groups. No gaps were observed in fifteen and thirty days; while bone implant contact (BIC), threads inner area bone (BAIT) and bone area outer threads (BAOT), were the most represented in the experimental sample. Additionally, there were no signs of bone resorption inflammation on either surface [61].

#### 6.3.4. Histological Evaluation

Through the optical microscopy all thirty-six implants appeared embraced by bone with a high degree of osseointegration; the lower threads with braided bone and bone marrow spaces, the upper coils in contact with cortical bone as shown in Table 5.

The results therefore confirmed a statistically significant difference in BIC, BAIT and BAOT between uncoated titanium and type I collagen-coated titanium. Type I collagen was used as a migration influence coating, adhesion, proliferation and differentiation of cells on titanium implants improving early osteogenesis but without formation of bone far from the surface of the same. The positive influence coating of titanium implants through the use of biomolecules was also confirmed in small pig’s models; in the experimental study, the authors found a greater correlation between implant and bone density increase [64,65,66].

## 7. Discussion

Thanks to new technologies it has been possible to recreate new, innovative surfaces, with chemical-physical modifications in order to accelerate osseointegration, with the aim of shortening the patients’ edentulous period and rehabilitation times. Furthermore, attacking biomolecules on the implant surface, such as bioactive compounds and multifunctional molecules, could promote the osteogenetic process around the implants, including induction of cell adhesion, osteogenic stimulation or even further antibacterial effects related to the management of peri-implantitis. Long-term clinical studies are still needed to compare the performance of different coatings and evaluate success rates. Furthermore, further studies should also examine whether traditional implant surface treatment and coating can achieve reliable therapeutic effects, especially in terms of achieving stability and osseointegration, as well as avoiding inflammation, infection, mobility and mechanical complications. In the future, more optimized modified coating technologies will be exploited to improve implant performance, which would be of great benefit to edentulous patients [67].

HA is a sulfur-free sulfur glycosaminoglycan without a protein core; it consists of long repeated sequences of two simple sugars such as glucuronic acid and N-acetylglucosamine. Both, with negative charges, when they bind together give rise to a linear molecule, very flexible and soluble. The great solubility is important to ensure the hydration of the tissues within which, in fact, it surrounds itself with water molecules to perform functions such as cushioning and lubrication [61,68].

In the field of aesthetic medicine, it has been used for several years to combat the skin aging by exploiting the mechanism of hydration through small local injections [18,69]. In addition to the recognized use of cosmetic treatments for years, biotechnologies have recently succeeded in developing a wide range of products for intraoral applications. These also include dental aesthetics [59].

The use of HA in periodontology for the closure of black interdental triangles with small gel infiltrations, directly into the gum, was definitely a great impact [70]. This molecule is used based on its chemical characteristics, such as hygroscopicity, viscoelastic and filling properties of space, bacteriostatic effect, anti-edema, antioxidant, biocompatible and non-immunogenic. In the dental field, a new frontier is certainly the use of HA as an adjuvant in healing processes after the insertion of titanium implants. Today, in fact, the implant application is considered the gold standard for replacement of missing teeth [56]. The introduction of osteointegrated implants in dentistry has been the cornerstone in clinical practice for the rehabilitation of partially or totally edentulous patients. The same expectations for prosthetic rehabilitation are always higher in terms of time, function and aesthetics and a new moment can be the evaluation of reducing biological healing times through the use of hyaluronic acid gel over the implant.

These results do not come as a result of a physical modification of the surface, as the micro-amplitude does not undergo changes through this addition of collagen given the thin nanometer-like thickness, but of a chemical change in the creation of the interface. HA, used as a coating, acts on migration, adhesion, proliferation and differentiation of cellular precursors on titanium implants improving the connection between implant and bone.

The improvement of the bioactivity of implant surfaces through the addition of HA could therefore facilitate the placement of early loading prosthetic products satisfying the patients’ requests [71,72].

New studies should be conducted to evaluate the functional load in dental implants with addition of HA to confirm that the bone-implant interface has a considerable advantage in the application of this gel [73].

The idea comes from the fact that the gel increases the amount of newly formed bone especially in the first weeks. The possibility of anticipating the growth curve of secondary stability, not waiting for the physiological decrease in primary stability, diagram of Tom Taylor [74], could be useful in the complete arch rehabilitative treatment of totally edentulous patients; thus, allowing the positioning of prosthetic products in a single session, shortening the times of biological healing and post-surgical bone remodeling. Bone function from early deceptive loading can also prevent excessive remodeling of the remaining crest as mastication mechanics would be a functional stimulus for homeostasis maintenance between osteoblasts and osteoclasts that do not rely solely on press fitting due to stability primary. HA, by interacting positively with TNF- α-cytokine involved in acute phase inflammatory reactions, would harden by the chemotactic action, an effect on other inflammatory cells resulting in stimulation of angiogenesis and recruitment of endogenous fibrogenic cells of the lesion thus promoting early osteogenesis. The formation of osteoid tissue and the presence of newly formed bone tissue between the implant coils testify to the role of HA as a positive application in the osseointegration process, thus favoring acceleration in the achievement of stability secondary early compared to the use of standard dental implants.

## 8. Conclusions

Unless otherwise proven, the addition of HA to the implant surface may also play a role in the soft tissue morphology. The use of collagen molecules can favor a maturation of the alveolar tissues avoiding the possible disadvantage of transparency and therefore of visibility of the implant body from the mucosa. The literature shows that the presence of these modified surfaces can not only favor the healing phases, but also play a role in the management of implant pathology. Certainly, further studies are needed to confirm what was found, on a large sample. The final hypothesis should be supported by experimental studies in animals and humans to evaluate the final results and confirm the initial idea.

## Figures and Tables

**Table 1:** Classification of titanium.

Alloy	Chemical Composition
Grade I	Ti (2.15 Fe; 0.12 O_2_)
Grade II	Ti (0.20 Fe; 0.18 O_2_)
Grade III	Ti (0.25 Fe; 0.25 O_2_)
Grade IV	Ti (0.30 Fe; 0.35 O_2_)
Grade V	Ti (0.06 Al; 0.04 V)

**Table 2:** Dental implant titanium alloys XRF (X-Ray Fluorescence) results.

**Grade V Dental Implant Sample**	Titanium (Ti) (91.9%) Aluminum (Al) (4.05%) Vanadium (V) (3.89%) Iron (Fe) (0.12%) Molibdenum (Mo) (<0.008%)

**Table 3:** TNF-α expression on both groups evaluated by the immunohistochemical tests.

	Control Group	Test Group
1 week	Antibody positivity for fibroblasts, osteoblasts and endothelial cells. Negativity for osteoid cells	Antibody positivity for fibroblast, osteoblast, osteocites, adipocites and endothelial cells. Negativity for osteoid cells
2 weeks	Bone marrow stem cells, osteoblasts, osteoclasts and osteocites antibody positivity. No bone trabeculature.	Bone marrow stem cells, osteoblasts, osteoclasts and osteocites antibody positivity. No bone trabeculature.
4 weeks	Bone marrow stem cells, osteoblasts, osteoclasts and osteocites antibody positivity. No new formation bone matrix.	Bone marrow stem cells, osteoblasts, osteoclasts and osteocites antibody positivity. No new formation bone matrix.

**Table 4:** Histomorphometric analysis.

Area	Control Group	Test Group
Bone	2697.7	3252.3
Bone with osteoid tissue	4704.1	5887.3

**Table 5:** BIC, BAIT and BAOT evaluation of both groups.

	Control Group	Test Group
15 days	Trabecular bone near to dental implant surface. Few inflammatory cells. Mean BIC 22.42 ± 4.5%, BAIT 23 ± 0.8%, BAOT 19 ± 0.8%	Trabecular bone near to dental implant surface. Numerous osteoblast in contact with dental implant surface. Mean BIC 27.5 ± 3.1%, BAIT 31 ± 0.8%, BAOT 21.8 ± 1%
30 days	Mature bone in contact with dental implant surface. Osteoblasts presence and absence of inflammatory exudate. Mean BIC 51.2 ± 3.9%, BAIT 28 ± 0.8%, BAOT 36 ± 0.8%	Mature bone with Haversian organization in contact with dental implant surface. Osteoblasts activity. Mean BIC 55.3 ± 3.2%, BAIT 39 ± 2.2%, BAOT 38 ± 2.2%
60 days	Mature bone in contact with dental implant surface. Osteoblasts activity. Mean BIC 53.32 ± 3.2%, BAIT 35 ± 2.3%, BAOT 36 ± 2.3%	Mature bone organization in contact with dental implant surface. Osteoblasts activity. Mean BIC 63.6 ± 2.9%, BAIT 42 ± 2.3%, BAOT 44 ± 2.3%

## Data Availability

All data are available on request to corresponding author.
